# Spatiotemporal dynamics of cyanobacterial blooms in the middle and lower reaches of the Xin River (Jiangxi, China) and their driving factors

**DOI:** 10.7717/peerj.21445

**Published:** 2026-08-06

**Authors:** Shiyu Zhou, Jinfu Liu, Jie Zhu, Guangshun Liu, Yezhi Huang, Yuwei Chen, Wei Li, Yu Xia, Liancong Luo

**Affiliations:** 1 Jiangxi Key Laboratory of Water Resources Allocation and Efficient Utilization, College of Water Conservancy, Jiangxi University of Water Resources and Electric Power, Nanchang, Jiangxi, China; 2 Jiangxi Key Laboratory for Intelligent Monitoring and Integrated Restoration of Watershed Ecosystem, School of Soil and Water Conservation, Jiangxi University of Water Resources and Electric Power, Nanchang, Jiangxi, China

**Keywords:** Xin River, Cyanobacteria, Spatiotemporal variation, Water environmental factors, Correlation analysis

## Abstract

This study analyzed phytoplankton monitoring data (2019–2023) from the middle-lower reaches of the Xin River to clarify cyanobacterial spatiotemporal distributions and their environmental drivers. Sixty-six phytoplankton genera across seven phyla were detected, with Chlorophyta being the dominant phylum, followed sequentially by Bacillariophyta and cyanobacteria. The inter-annual variation of phytoplankton biomass ranged from 2.42 to 11.46 mg/L, while the cyanobacteria biomass varied from 0 to 2.63 mg/L, accounting for 7.54% of the total phytoplankton biomass. The dominant cyanobacteria species included * Microcystis*, * Planktothrix*, and members of Phormidioideae, with the dominant species showing some heterogeneity across different regions. For the study area, the average River Trophic Status Index (RSI) was recorded as 54.89, indicating seasonal water quality variations: favorable during the dry season; slightly polluted in most river sections during the rising period; moderately polluted in most sections during the wet period; and generally slightly polluted during the rece ssion period. Statistical analyses (Pearson correlation and redundancy analysis (RDA)) revealed that cyanobacteria and their dominant species had significant correlations (*p* < 0.05) with environmental factors including total phosphorus (TP), orthophosphate (PO_4_^3−^-P), dissolved total nitrogen (DTN), chemical oxygen demand (COD_Mn_), Secchi Depth (SD), were the primary environmental factors driving the spatiotemporal distribution of cyanobacteria. This study provides for the first time an earlier seasonal emergence of cyanobacteria in lake entrance areas, addresses the domestic gap concerning inadequate attention to small and medium-sized rivers (*e.g.*, the Xin River) relative to large lakes such as Taihu Lake and Chaohu Lake and holds crucial reference value for early warning systems aimed at preventing cyanobacterial encroachment into Poyang Lake.

## Introduction

River ecosystems provide critical ecological services in terms of providing habitats for organisms, regulating climate, and mediating biogeochemical cycling (Xi et al., 2022). However, with the development of industry and agriculture and the growth of population, rivers are facing severe nitrogen and phosphorus pollution. Agricultural fertilizer runoff, urban domestic sewage, livestock and poultry wastewater, and point-source discharges from specific industries such as food processing and papermaking are the main pathways leading to excessive inputs of nitrogen and phosphorus nutrients into rivers (Carpenter et al., 1998; Howarth, Sharpley & Walker, 2002; Van Drecht et al., 2009; Min et al., 2021; Meng, Su & Zheng, 2004). Such excessive nutrient loading has triggered global eutrophication, resulting in frequent cyanobacterial blooms (Watson et al., 2016; Zhang & Kong, 2015; Ping, 2015; Liu et al., 2014). Cyanobacterial blooms not only reduce the utilization value of water resources but also disrupt aquatic ecological balance by inhibiting photosynthesis of submerged macrophytes and causing dissolved oxygen depletion (Havens, 2008; Shao et al., 2024; Rabalais, 2002). In urban waters, severe algal blooms can also block water supply filtration systems and pose public health risks (Li et al., 2018; Huisman et al., 2018). Therefore, exploring the driving mechanisms of cyanobacterial blooms has become an urgent topic in global ecological and environmental research (Dai et al., 2023; Mu, Huang & Zhang, 2013).

International research has long emphasized the non-linear effects of temperature on cyanobacterial growth. Paerl & Huisman (2008) suggested that the optimal temperature range for cyanobacteria typically falls between 20–30 °C, with their competitive advantage becoming significantly enhanced under high-temperature conditions. Regarding light, Jöhnk et al. (2008) investigated photoadaptive mechanisms in cyanobacteria—such as phycobilisome regulation—along with their respective photoinhibition thresholds. The role of the nitrogen-to-phosphorus ratio in regulating cyanobacterial blooms has also been widely examined; Schindler (2012) emphasized the importance of phosphorus limitation in controlling eutrophication. However, Conley et al. (2009) indicated that global warming may reduce the effectiveness of dual nitrogen and phosphorus control strategies. In China, studies on cyanobacterial blooms have largely concentrated on large and medium-sized lakes such as Taihu and Chaohu. Li et al (2023) identified a strong correlation between bloom outbreaks in the middle-lower Yangtze River regions and both the rate of spring warming and the duration of summer high temperatures. In terms of nutrient dynamics, Wang et al. (2024) proposed that after external nutrient inputs were reduced in Lake Chaohu, internal nutrient release became the dominant factor influencing bloom formation. Recent studies have increasingly highlighted the interactive effects of multiple environmental factors. For example, Sarpong et al.’s (2020) research team used modeling approaches to demonstrate that rising temperatures and increased nutrient concentrations act synergistically to promote an exponential rise in cyanobacterial biomass, while reduced wind speed exacerbates ecological damage by prolonging bloom persistence.

The Xin River ranks among the five major rivers in Jiangxi Province and is geographically positioned in the northeastern part of the province, between 117°E and 119°E in longitude and 27°N and 29°N in latitude. It originates from Yujing Peak in the Huaiyu Mountains on the border of Zhejiang and Jiangxi, and near Daxidu in Yugan County, it splits into two branches: a western branch that flows into Poyang Lake *via* Ruihong in Yugan County, and an eastern branch that enters the Rao River *via* Zhuhu Mountain. The Xin River basin spans multiple counties and districts, including Yushan, Guangfeng, Shangrao, Yanshan, Yugan, Xinzhou, and Yuehu, making it the most important water source for the region. However, with rapid societal development, water pollution issues have become increasingly prominent in the basin. Industrial wastewater from businesses, domestic sewage from residents, and uncontrolled sand mining in the river have all severely degraded the water quality (Bao & Ye, 2023). Therefore, investigating the ecological environment and water quality of the Xin River is of paramount importance. Current research on cyanobacterial blooms has largely focused on large and medium-sized lakes. For example, Hu et al. (2024) conducted a specific study on the spatiotemporal distribution characteristics and driving factors of cyanobacteria in Poyang Lake, and Yang et al. (2019) explored various causes of cyanobacteria outbreaks in Lake Taihu. In contrast, there has been insufficient attention paid to small and medium-sized rivers like the Xin River, leaving the unique mechanisms driving cyanobacterial blooms in its basin unclear. Furthermore, the cascade response mechanism of “hydrology-nutrients-algae” in the context of climate change warrants further investigation. Based on *in situ* field sampling and continuous monitoring, this study investigates the spatiotemporal dynamics of cyanobacteria in the middle-lower reaches of the Xin River and examines the linkages between cyanobacteria and key environmental factors, with a particular focus on nutrients. The objective of this study is to offer a scientific foundation and reference for the control and prevention of eutrophication and cyanobacterial blooms in the Xin River basin and the main area of Poyang Lake (Baird, Eaton & Rice, 2017).

## Materials and Methods

### Sample collection and lab analysis

**Figure 1:**
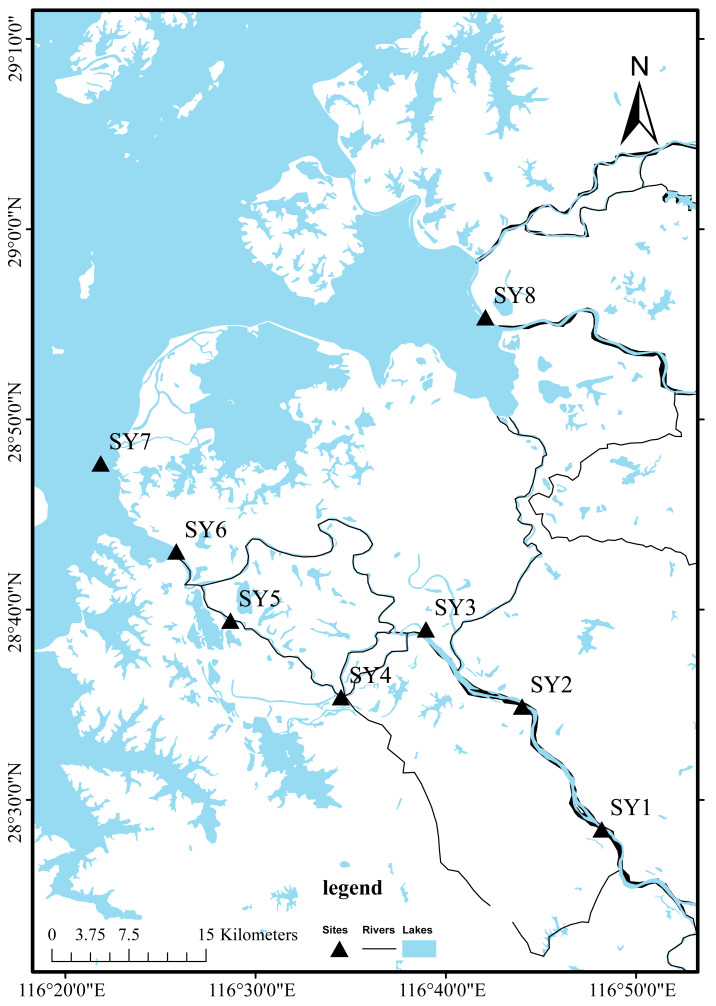
Location of sampling sites in the middle-lower reaches of Xin River.

To explore the spatiotemporal distribution characteristics of cyanobacterial blooms and their driving factors in the middle-lower reaches of the Xin River, from 2019 to 2023, this study performed quarterly ecological field sampling at eight sampling sites (SY1–SY8 for short) located in the study area. Annual sampling was implemented in January (dry season), April (rising season), July (wet season), and October (recession season), spanning a 5-year observational period. In addition, based on the habitat heterogeneities across different sub-regions within the middle-lower reaches of the Xin River, the study area was divided into two distinct zones: the main stream zone, which includes SY1 (Huangjinbu), SY2 (Longjin Bridge), SY3 (Hebu Bridge), SY4 (Fenggang), SY5 (Zhehu), and SY6 (Ruihong Bridge); and the lake inlet zone, which comprises SY7 (Xin River Estuary) and SY8 (East Branch Estuary of the Xin River). The specific distribution of these sampling sites is illustrated in Fig. 1.

*In situ* determination of water quality parameters was conducted using a YSI 6600V2 multi-parameter water quality sonde. After the displayed readings achieved stability, the following parameters were recorded: water temperature (WT), pH, dissolved oxygen (DO), electrical conductivity (EC), turbidity (Turb), oxidation–reduction potential (ORP), and total dissolved solids (TDS). Secchi disk measurements were used to determine Secchi Depth (SD), and a handheld ultrasonic depth sounder was employed for the measurement of water depth (WD). The concentration of Chl *a* was quantified by the hot ethanol extraction spectrophotometric method (Chen, Chen & Hu, 2006). For nutrient parameters, including total nitrogen (TN), total phosphorous (TP), NH_4_^+^-N, NO_3_^−^-N, and orthophosphate (PO_4_^3−^-P), measurements were performed in compliance with the Technical Specifications for Eutrophication Investigation (Jin & Tu, 1990). All physical-chemical indicators and ecological indicators related to the aquatic ecosystem in this study were analyzed and determined strictly following national standard methods at the State Key Laboratory of Lake Science and Environment.

### Phytoplankton analysis

Equal volumes of water samples were collected from the surface, middle, and bottom layers of the water body using a 5 L plexiglass water sampler. After the water samples were mixed uniformly, 1 L was sealed and brought back to the laboratory for phytoplankton analysis. Phytoplankton samples were preserved in 10 mL of Lugol’s solution and subjected to a 48-hour sedimentation period prior to microscopic identification and enumeration, then the sample was siphoned to a 30 mL final volume and thoroughly mixed. A 0.1 mL subsample was then aliquoted into a counting chamber for analysis under an upright optical microscope (40 × magnification) across 100 random fields, each sample underwent two independent counting iterations. The reliability of the enumeration was validated only if the relative difference between the two replicates and their average was within a ±15% threshold (Hu & Wei, 2006). After identification, the phytoplankton biomass was calculated based on the measured cell volume of the phytoplankton (Hillebrand et al., 1999).

### Data analysis

To determine the dominance of cyanobacteria, the dominance index (Y) was employed, and its calculation formula is given as follows:

\begin{eqnarray*}\mathrm{Y}= \frac{{\mathrm{N}}_{\mathrm{i}}}{\mathrm{N}} \times {\mathrm{f}}_{\mathrm{i}}. \end{eqnarray*}

For this formula, N_i_ denotes the total number of individuals of a specific taxon in the sample; N signifies the total number of individuals of all organisms within the sample; *f*_*i*_ refers to the occurrence frequency of a given species. A species was regarded as a dominant genus when Y > 0.02 (Hao et al., 2014).

The trophic state of the water body in the middle-lower reaches of the Xin River was evaluated using the River Trophic Status Index (RSI)—a method under the multi-parameter comprehensive evaluation framework (Zhang et al., 2006). The calculation formula for RSI is defined as follows:

\begin{eqnarray*}\mathrm{RSI}(\sum )=\sum _{\mathrm{j}=1}^{\mathrm{n}}{\mathrm{W}}_{j}\times \mathrm{RSI}(\mathrm{j}). \end{eqnarray*}

In the formula, *RSI* (∑) denotes the comprehensive River Trophic Status Index, RSI(j) represents the trophic status index of the j-th parameter, and *W*_*j*_ is the normalized correlation weight of the trophic status index for the j-th parameter.

The trophic state of the river was classified using a numerical scale ranging from 0 to 100. Table 1 provides the correspondence between water quality categories and their respective RSI scores.

**Table 1:** Classification of river trophic status index.

Classification of river trophic state index	Score value RSI (Σ)	Qualitative evaluation
Oligotrophic	0 <RSI(Σ) ≤30	Excellent
Mesotrophic	30 <RSI(Σ) ≤50	Favorable
Lightly Eutrophic	50 <RSI(Σ) ≤60	Light Pollution
Moderately Eutrophic	60 <RSI(Σ) ≤70	Moderate Pollution
Heavily Eutrophic	70 <RSI(Σ) ≤100	Severe Pollution

In this study, data were initially organized and analyzed using Excel. The interannual variation trends and spatial distribution patterns of phytoplankton in the middle-lower reaches of the Xin River, along with the spatiotemporal dynamics of cyanobacteria biomass and abundance, were visualized *via* box plots, bar charts, line charts, and chord diagrams using Origin 2024. The sampling site map was generated using ArcMap 10.8, and redundancy analysis (RDA) was performed with Canoco 5.0. Within Canoco 5.0, detrended correspondence analysis (DCA) was first conducted on the total biomass of cyanobacteria and the data of its dominant species. The results showed that the maximum gradient length was less than 3, so redundancy analysis (RDA) was selected. For environmental and species data, except for pH values, all were transformed using the lg(x+1) method (Shi et al., 2008) to approximate a normal distribution (Flores & Barone, 1998). For environmental factors, Monte Carlo permutation tests were used to screen out significantly influential factors (*P* < 0.05), which were then used for RDA.

## Result

### Physicochemical characteristics

During the study period, the mean water temperature concentration in the middle-lower reaches of the Xin River demonstrated a consistent seasonal dynamic, characterized by an initial increase followed by a subsequent decrease: both parameters gradually rose from their lowest values in the dry season (January), peaked in the wet season (July), and subsequently began to decline. In terms of seasonal variation trends, SD, chemical oxygen demand (COD_Mn_), and TP showed no obvious fluctuations and remained relatively stable. Specifically, COD_Mn_ met the Class II criteria specified in the Environmental Quality Standards for Surface Water (GB 3838-2002) across all four hydrological seasons. The overall concentration of TP was relatively low, with the minimum value of 0.052 ± 0.34 mg/L observed in the dry season, which also complied with the Class II standards of GB 3838-2002. The concentrations of TN, NH_3_-N, and NO_3_^−^-N generally presented a decreasing trend, with their concentrations gradually declining with seasonal changes and reaching the lowest levels in the wet season. A slight increase in concentrations was observed starting from the recession season (October). For TN, higher concentrations were detected in the dry season (January) and rising season (April), which were classified as worse than Class V according to GB 3838-2002; in the wet season and receding water season, TN concentrations fell into the Class IV category. As for NH_3_-N, it met the ClassII standards in the dry season and rising water season, and further reached the Class I standards in the wet season and receding water season (Table 2).

**Table 2:** Changes of environmental factors in Xin River.

Physicochemical parameters	Dry season	Rising season	Wet season	Recession season
WT (°C)	10.1 ± 4.9	20.6 ± 3.8	30.3 ± 3.9	23.1 ± 2.7
pH	7.35 ± 1.46	8.39 ± 1.8	7.53 ± 0.97	7.04 ± 2.77
SD (cm)	0.8 ± 0.6	0.7 ± 0.4	0.6 ± 0.4	0.6 ± 0.4
TN (mg/L)	2.32 ± 0.56	1.75 ± 0.95	1.23 ± 0.75	1.27 ± 0.46
NH_3_-N (mg/L)	0.25 ± 0.21	0.18 ± 0.14	0.09 ± 0.07	0.11 ± 0.09
NO_3_^−^-N (mg/L)	0.83 ± 0.76	0.54 ± 0.32	0.33 ± 0.32	0.34 ± 0.23
TP (mg/L)	0.052 ± 0.034	0.063 ± 0.023	0.062 ± 0.041	0.068 ± 0.043
PO_4_^3−^-P (mg/L)	0.016 ± 0.013	0.019 ± 0.017	0.009 ± 0.006	0.020 ± 0.017
COD_Mn_ (mg/L)	2.4 ± 0.8	2.9 ± 1.1	2.5 ± 1.3	2.7 ± 0.9
Chl *a* (μg/L)	4.08	7.62	13.73	9.16

### Evaluation of water trophic status

**Figure 2:**
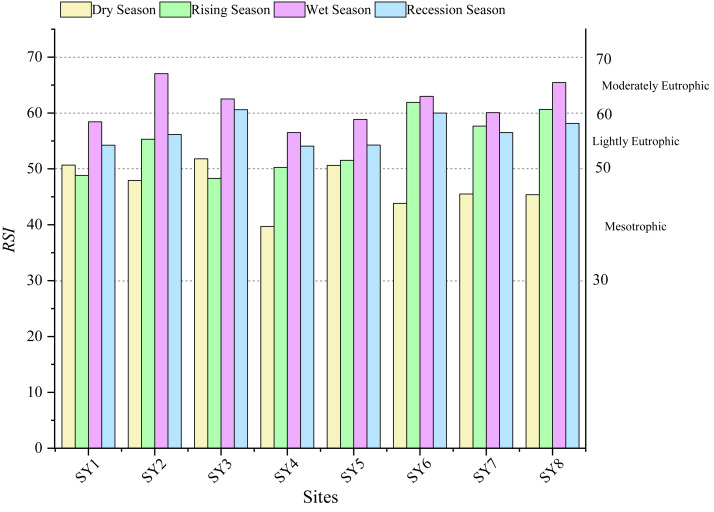
Comprehensive River Trophic Status Index in the middle-lower reaches of Xin River.

As shown in Fig. 2, for the River Trophic Status Index (RSI) of the Xin River, all sampling sites except SY1 and SY3 had RSI values ranging from 30 to 50 during the dry season. This indicates that the water quality in the study area was generally at the mesotrophic level in the dry season, with a qualitative water quality assessment of “good”. In the wet season, RSI values of most sites exceeded 60, except for SY1, SY4, and SY5 (with RSI ranging from 50 to 60). Consequently, the water quality of most sections in the study area fell into the mesotrophic-eutrophic level during the wet season, corresponding to a qualitative assessment of “moderate pollution”. Notable differences in trophic state were observed across the four hydrological seasons. Overall, RSI values were lower in the dry and rising water seasons, and highest in the wet season—reflecting the pattern that trophic levels were higher in summer and autumn than in spring and winter. Additionally, RSI values of most sampling sites showed a gradual increase from the dry season to the wet season across all four periods, which confirms that the eutrophication degree of the water body in the study area was more severe during the wet season. From a spatial perspective, the eight sites in study area generally exhibited higher RSI values at both ends and lower values in the middle. This indirectly suggests that urbanization in the middle area of the Xin River and its lake entrance areas has progressed more rapidly, leading to greater discharge of industrial and domestic sewage compared to other sampling locations. In summary, the water body in the middle-lower reaches of the Xin River was generally at the lightly eutrophic level, with significant water pollution observed in some river sections, resulting in an overall qualitative water quality assessment of “light pollution”.

### Spatiotemporal characteristics of phytoplankton community

In the phytoplankton samples collected from the middle and lower reaches of the Xin River during this survey, a total of 66 genera belonging to seven phyla were identified, namely Cyanobacteria, Bacillariophyta, Chlorophyta, Euglenophyta, Dinophyta, Cryptophyta, and Chrysophyta.

**Figure 3:**
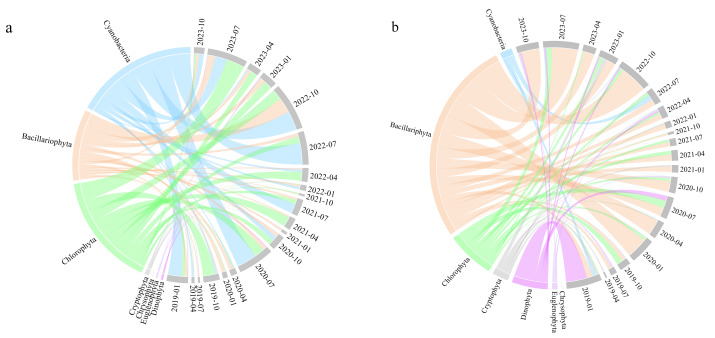
Chord chart of annual variations in phytoplankton in the main stream of Xin River. (A) Phytoplankton abundance, (B) Phytoplankton biomass.

**Figure 4:**
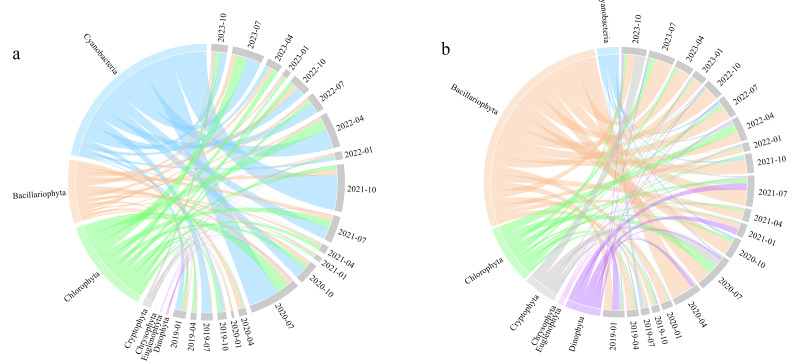
Chord chart of annual variations in phytoplankton in the lake entrance areas of Xin River. (A) Phytoplankton abundance, (B) phytoplankton biomass.

Phytoplankton biomass and abundance in the main stream of the Xin River and its lake entrance area were predominantly contributed by Bacillariophyta and cyanobacteria, with respective contributions of 31.81% and 19.48% in the main stream, and 29.11% and 27.82% in the lake entrance area. From 2019 to 2023, the total phytoplankton biomass in the study area of the Xin River ranged between 2.42 mg/L and 11.46 mg/L. Peak values were observed in the summers of 2020, 2021, and 2023, reaching 11.46 mg/L, 9.42 mg/L, and 8.04 mg/L, respectively. However, distinct patterns were recorded in 2019 and 2022, during which biomass peaks occurred in winter and spring, with values of 6.54 mg/L and 7.94 mg/L, respectively. Significant seasonal variations in phytoplankton biomass were identified in both the main stream and the lake entrance area. Except in 2019, biomass during summer (main stream: 1.95 mg/L; lake entrance: 3.45 mg/L) and autumn (main stream: 1.82 mg/L; lake entrance: 2.27 mg/L) exceeded that in spring (main stream: 1.41 mg/L; lake entrance: 2.01 mg/L) and winter (main stream: 1.04 mg/L; lake entrance: 1.07 mg/L) across the other four years. Furthermore, the lake entrance area exhibited significantly higher phytoplankton biomass than the main stream throughout the study period. Phytoplankton abundance during this period ranged from 2.23 × 10^6^ cell/L to 20.75 × 10^6^ cell/L. Mirroring the seasonal pattern observed for biomass, phytoplankton abundance was higher in summer and autumn relative to winter and spring across all study years, except in 2022. Additionally, the total phytoplankton abundance in 2019 (16.68 × 10^6^ cell/L) was significantly lower than that in the other four years (34.88 × 10^6^ cell/L) (Figs. 3 and 4).

**Figure 5:**
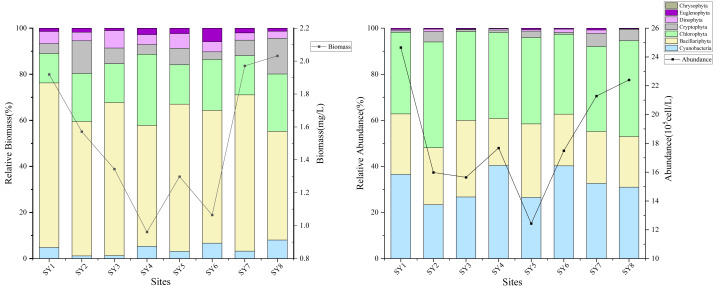
Spatial distribution of phytoplankton abundance and biomass in the middle-lower reaches of the Xin River.

From a spatial perspective (Fig. 5), the lowest phytoplankton biomass was recorded at sampling site SY4, followed by SY6 and SY5. While these three sites are located centrally among the eight sampling stations, the maximum phytoplankton biomass was observed at stations SY1 and SY8. Overall, phytoplankton biomass in the study area exhibited a pattern of being higher at both ends and lower in the middle segment. The spatial distribution of phytoplankton abundance was similar to that of biomass, with higher abundance at the two end segments and lower abundance in the middle. When considering phytoplankton biomass and abundance across different habitats, the main stream of the Xin River showed a decreasing trend from upstream to downstream, while a sharp increase was observed at the lake entrance.

### Spatiotemporal characteristics of cyanobacteria species

**Figure 6:**
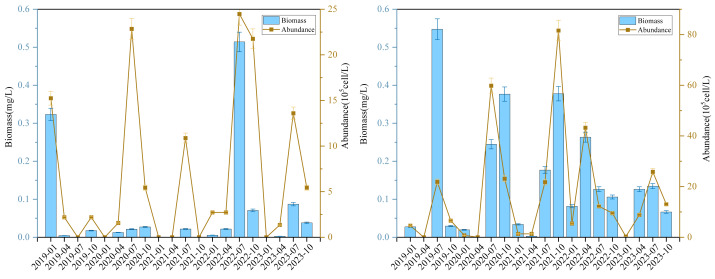
Time variation of cyanobacteria. (A) Main stream, (B) lake entrance areas.

From a temporal perspective (Fig. 6), cyanobacteria biomass and abundance in both the main stream and lake entrance areas of the Xin River’s middle and lower reaches exhibited substantial fluctuations throughout the year, with distinct annual and seasonal variation patterns. Notably, the variation trends of cyanobacteria biomass and abundance were nearly consistent between the two areas. In the main stream area, *Microcystis* and *Planktothrix* were the dominant cyanobacterial species in both spring and autumn. During summer, the dominant taxa included *Microcystis*, *Anabaena*, *Planktothrix*, and *Phormidioideae*. By contrast, *Microcystis* was the sole dominant cyanobacteria in winter. And cyanobacteria biomass and abundance were higher in summer and autumn than in spring and winter, with the only exception being 2019—when peaks occurred in winter (0.32 mg/L for biomass and 15.25 × 10^5^ cell/L for abundance). Among all surveyed years, the highest cyanobacteria biomass and abundance in the main stream were recorded in summer 2022, reaching 0.52 mg/L and 24.48 × 10^5^ cell/L respectively (the highest values in the five-year period).

In the lake entrance area, the peaks of cyanobacteria biomass and abundance occurred in summer 2019 (0.55 mg/L) and autumn 2021 (81.6 × 10^5^ cell/L) respectively. Collectively, the variation trends of cyanobacterial biomass and abundance in the lake entrance area showed high similarity to those in the main stream, and both also demonstrated higher values in summer and autumn relative to winter and spring. However, a notable deviation was observed in spring 2022: cyanobacteria biomass and abundance in this season reached the annual peak (0.26 mg/L and 43.25 × 10^5^ cell/L) and were significantly greater than those observed in the other three seasons within the same year. Additionally, cyanobacteria biomass in spring 2023 was already close to that in summer of the same year. These observations suggest that the growth and bloom of cyanobacteria in the lake entrance area may be advancing in timing across years.

**Figure 7:**
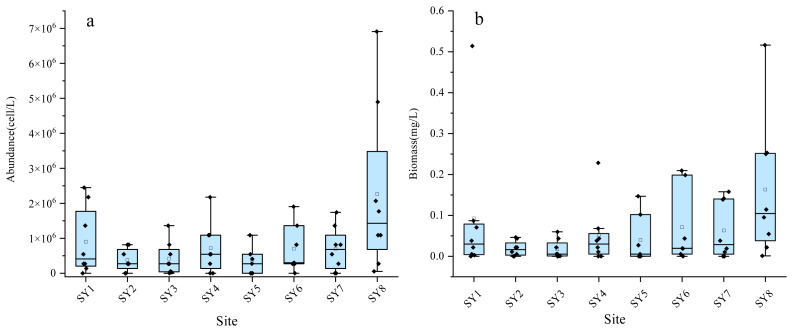
Spatial changes of cyanobacteria. (A) Cyanobacteria abundance, (B) cyanobacteria biomass.

Spatially (Fig. 7), the biomass and abundance of cyanobacteria in the study area of the Xin River generally showed a trend wherein the lake entrance had higher values compared to the main stream.

At the specific sampling site level, cyanobacteria biomass and abundance were higher at SY1 (located in the middle reaches) and SY8 (located in the lower reaches) compared to the other sites in the middle segment of the sampling range—exhibiting a “higher at both ends, lower in the middle” distribution. Additionally, there were no significant differences in cyanobacteria biomass and abundance among the various sampling sites within the main stream. In contrast, cyanobacteria biomass and abundance at the lake entrance sites were significantly different from those in the main stream (*p* < 0.05).

This observation indicates that there are substantial differences in cyanobacteria habitats between the main stream and the lake entrance areas of the Xin River, with cyanobacteria in the study area being more concentrated at the estuary where the Xin River flows into Poyang Lake.

**Figure 8:**
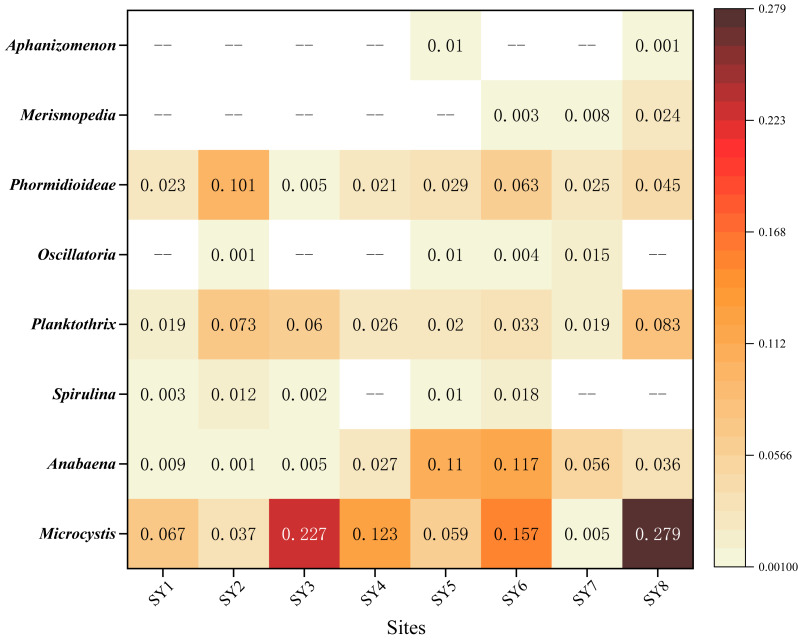
Dominant species and dominance index of cyanobacteria.

Throughout the survey period of this study, a total of seven dominant genera within the phylum cyanobacteria were identified (Fig. 8). These genera are *Microcystis*, *Anabaena*, *Spirulina*, *Planktothrix*, *Oscillatoria*, *Phormidioideae*, and *Merismopedia*. The assemblage of dominant cyanobacterial genera differed across distinct habitats in the study area, Within the main stream, sampling sites SY4, SY5, and SY6 each harbored four dominant cyanobacterial genera; site SY2 supported three dominant genera; and sites SY1 and SY3 had the lowest diversity of dominant genera, with only two each; At the lake entrance, site SY7 contained two dominant cyanobacterial genera, while site SY8 had the highest number of dominant genera among all lake entrance sites, totaling five.

And in the main stream, *Microcystis* exhibited the highest dominance at sites SY1, SY3, SY4, and SY6, with corresponding dominance index (Y) values of 0.067, 0.227, 0.123, and 0.157, respectively. At site SY2, *Planktothrix* was the dominant genus, with a Y-value of 0.073, while at site SY5, *Anabaena* showed the greatest dominance, with a Y-value of 0.11; At the lake entrance: *Anabaena* was the dominant genus at site SY7 (*Y* = 0.056), whereas *Microcystis* achieved the highest dominance at site SY8, with a Y-value of 0.279—the highest single-genus dominance recorded across all sampling sites.

Overall, the dominant genera of cyanobacteria in both the main stream and lake entrance areas were consistently dominated by four core genera: *Microcystis*, *Phormidioideae*, *Anabaena*, and *Planktothrix*. This shared dominance pattern highlights the ecological adaptability of these genera to the environmental conditions in Xin River, while site-specific variations in dominant genera reflect the influence of localized habitat characteristics.

### Relationship between cyanobacteria and environmental factors

**Figure 9:**
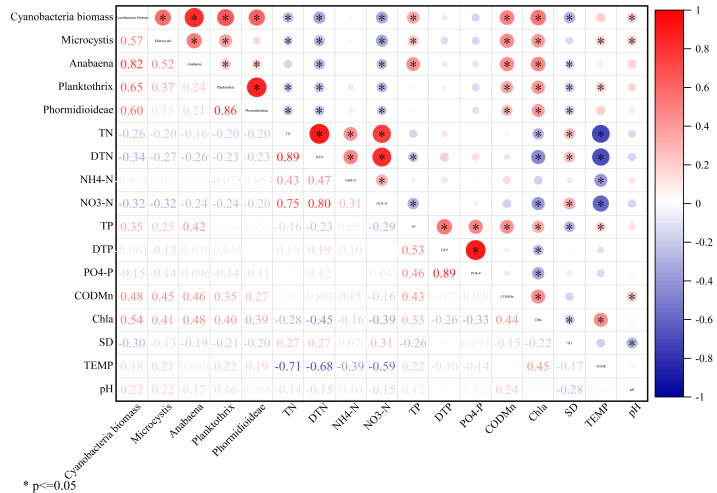
Correlation analysis between cyanobacteria and their dominant species in Xin River and environmental factors.

For the purpose of exploring the associations between cyanobacteria and water environmental factors in the middle-lower reaches of the Xin River, Pearson correlation analysis was carried out to examine the relationships between the biomass of cyanobacteria (including selected dominant cyanobacterial genera) and the surveyed aquatic environmental factors in the study area. To ensure the comprehensiveness and scientific rigor of the analysis results, the dataset used for this correlation analysis included the biomass of cyanobacteria and its selected dominant genera, paired with corresponding water environmental factor data, all extracted from the five-year (2019–2023) ecological monitoring dataset (Fig. 9).

The biomass of cyanobacteria and some of their dominant species showed negative correlations with TN, dissolved total nitrogen (DTN), NO_3_^−^-N, PO_4_^3−^-P, and Secchi Depth (SD), while exhibiting positive correlations with TP, COD_Mn_, WT, Chl*a*, and pH. Specifically, there was a significant negative correlation between TN and *Microcystis*, *Planktothrix*, and *Phormidioideae*, while showing an extremely significant negative correlation with the entire phylum cyanobacteria; TP exhibited a significant positive correlation with the phylum cyanobacteria and the genus *Microcystis*, while showing an extremely significant positive correlation with the genus *Anabaena*; NO_3_^−^-N was significantly negatively correlated with *Anabaena*, *Planktothrix*, and *Phormidioideae*, and extremely significantly negatively correlated with cyanobacteria and *Microcystis*; SD exhibited a significant negative correlation with *Anabaena* and *Planktothrix*, and a highly significant negative correlation with cyanobacteria. WT was significantly positively correlated with *Microcystis* and *Planktothrix*; pH was significantly positively correlated with cyanobacteria and *Microcystis*; Meanwhile, COD_Mn_ and Chl*a* were extremely significantly positively correlated with both cyanobacteria and all its dominant species.

**Table 3:** Redundancy analysis results of cyanobacteria and environmental factors in Xin River.

Watershed	Item	Characteristic value	Species-environment correlation	Cumulative percentage (%)	
				Species	Species-environment correlation
Main stream	Axis 1	0.4407	0.8115	44.07	80.52
	Axis 2	0.0941	0.6088	9.41	97.71
Lake entrance areas	Axis 1	0.2612	0.7345	26.12	78.23
	Axis 2	0.0496	0.484	4.96	93.09

Data of cyanobacteria and dominant species from the main stream and lake inlets were selected for Detrended Correspondence Analysis (DCA). The results showed that the lengths of the gradient of the first axis were all less than 3. Therefore, redundancy analysis (RDA) was employed to examine the relationships between environmental drivers (pH, SD, WT, DTN, TP, PO_4_^3−^-P, COD_Mn_, NO_3_^−^-N, Chl*a*) and response factors (cyanobacteria biomass, dominant species biomass) in the two regions (main stream and lake inlets) respectively. According to Table 3, the eigenvalues of Axis 1 and Axis 2 for the main stream are 0.4407 and 0.0941, respectively; the correlation coefficients between species and environmental factors are 0.8115 for Axis 1 and 0.6088 for Axis 2. For the lake inlet, the eigenvalues of Axis 1 and Axis 2 are 0.2612 and 0.0496, respectively; the correlation coefficients between species and environmental factors are 0.7345 for Axis 1 and 0.484 for Axis 2. The first two axes cumulatively explain 53.49% of species information and 97.71% of species-environment relationship information for the main stream, and 31.08% of species information and 93.09% of species-environment relationship information for the lake inlet. The ordination results are reliable.

**Figure 10:**
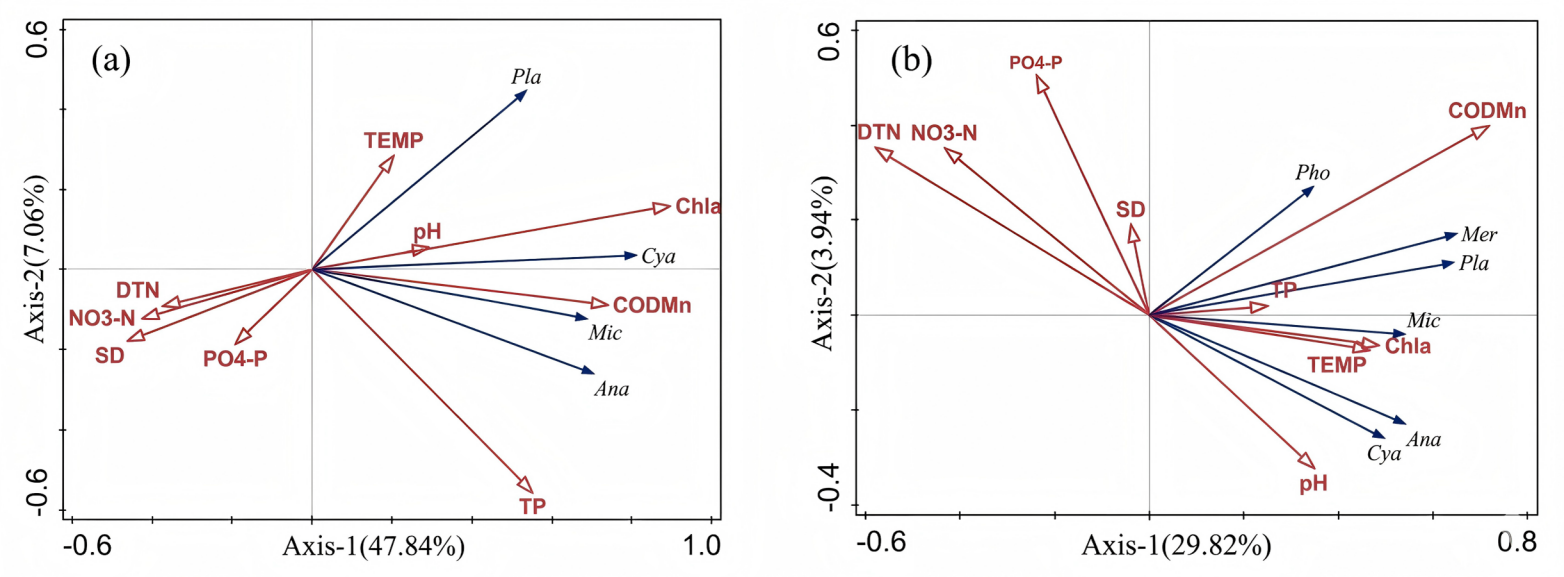
Redundancy analysis of cyanobacteria and dominant species and environmental. (A) Main stream, (B) lake entrance areas.

From the RDA results (Fig. 10) involving dominant species biomass, cyanobacterial biomass, and nine aquatic environmental factors, it can be observed that the biomass of each dominant species and the cyanobacteria biomass in the main stream exhibit a positive correlation with WT, pH, TP and COD_Mn_ while negatively correlated with other factors. Among these factors, Chl *a*, TP, and PO_4_^3−^-P show an extremely significant correlation with the distribution of the cyanobacterial community in this region (*p* < 0.01), and COD_Mn_ and SD exhibit a significant correlation with the distribution of the cyanobacterial community in this region (*p* < 0.05).

In the estuary area entering the lake, the cyanobacterial biomass and *Anabaena* biomass are negatively correlated with DTN, SD, NO_3_^−^-N, and PO_4_^3−^-P, and positively correlated with pH, WT, and COD_Mn_. *Microcystis* and *Phormidium* biomass showed contrasting correlation patterns, being positive with pH, SD, WT, and COD_Mn_, but negative with NO_3_^−^-N, PO_4_^3−^-P, and DTN. Among the environmental factors, DTN and COD_Mn_ display an extremely significant correlation with the distribution of the cyanobacterial community in this region (*p* < 0.01).

## Discussion

### Distribution characteristics of cyanobacteria in Xin River

In the study area of the Xin River, the biomass of cyanobacteria ranges from 0 to 2.63 mg/L, and their abundance ranges from 0 to 3.13  ×  10^7^ cells/L. High-density cyanobacterial cells are mainly concentrated in the Ruihong Town area of the main stream and the Xin River Estuary area of the lake entrance, which is consistent with the research results of Zou (2024) on the spatial distribution characteristics of phytoplankton in the middle-lower reaches of the Xin River.

In this study, among the main taxonomic groups composing the phytoplankton in the Xin River, Chlorophyta and Bacillariophyta account for more than 70% of the total algae, and the water ecological type is a Chlorophyta-Bacillariophyta type water body. However, this study found that the proportion of cyanobacteria exceeded 40% in some survey areas during certain quarters, and the water body was in a state of mild eutrophication or moderate eutrophication. Studies have shown that when the water exchange volume of a water system is large, the phytoplankton community in the water body is usually a community of Bacillariophyta and Chlorophyta; when the water exchange volume is small, it will change to a community of cyanobacteria and Bacillariophyta (Zhu, Wan & Zhao, 2010). The hydrodynamic conditions in the Xin River are complex and changeable, and the areas close to the shore are extremely vulnerable to the impact of human activities. This also explains why the Xin River, which is considered a mesotrophic-oligotrophic Chlorophyta-Bacillariophyta type water body among rivers and lakes (Paerl & Huisman, 2008; Paerl & Paul, 2012), has cyanobacterial blooms occurring in multiple sections of its study area. Also, the dominant species (*Anabaena* and *Microcystis*) in the Xin River of this study are commonly found in eutrophic water bodies, which also indicates to a certain extent that the water quality of the middle-lower reaches of the Xin River has been in a state of eutrophication.

In addition, as water temperature is one of the important influencing factors in the growth and reproduction of phytoplankton (Li et al., 2019), a relatively high temperature can affect the activity of enzymes in phytoplankton cells and their metabolic processes. This effectively improves the efficiency of cyanobacteria in absorbing light and enhances their photosynthetic capacity (Noyma et al., 2021; Pniewski & Sylwestrzak, 2018). The optimal temperature range for the growth and reproduction of most cyanobacteria is 25 °C to 35 °C (Huisman et al., 2018; Nalewajko & Murphy, 2001), while the growth of cyanobacteria will be restricted when the temperature is below 15 °C (Robarts & Zohary, 1987). Therefore, the relatively high water temperature in summer is conducive to the growth of cyanobacteria, enabling them to become the dominant species. In this study, the average water temperatures of the Xin River during the four hydrological periods (dry season, rising season, wet season, and recession season) were 10.1 °C, 20.6 °C, 30.3 °C, and 23.1 °C, respectively. Water temperature during the high-flow period is conducive to cyanobacterial growth; therefore, cyanobacterial blooms in the Xin River study area primarily occur in the high-flow period at selected monitoring sections.

### Response relationship between cyanobacteria and water environmental factors

The key environmental factors influencing cyanobacterial distribution vary across different regions (Zhang et al., 2013). In this study, the RDA results indicated that TP, PO_4_^3−^-P, COD_Mn_, and SD are the primary environmental factors affecting the distribution of cyanobacterial communities in the main stream of the middle and lower reaches of the Xin River. In contrast, DTN and COD_Mn_ are the key environmental factors influencing cyanobacterial community distribution in the lake entrance area of Xin River. Therefore, nitrogen and phosphorus nutrients are the dominant environmental factors regulating cyanobacterial community distribution in the middle-lower reaches of the Xin River, which is consistent with the findings of Gou et al. (2015). Meanwhile, nitrogen and phosphorus are also primary components of aquatic nutrients—essential for phytoplankton growth and reproduction—and serve as pivotal environmental factors influencing phytoplankton distribution patterns (Lope et al., 2009).

Studies have shown that algal blooms may occur when the concentrations of nitrogen and phosphorus in water reach 0.2 mg/L and 0.025 mg/L, respectively (Birch & WcCaskie, 1999). In the study area of Xin River, the average concentrations of nitrogen and phosphorus across the four hydrological periods are far higher than these threshold values for algal blooms. Notably, the regions with relatively high nitrogen and phosphorus levels are mainly distributed between sampling sites SY6 and SY8. This river section is adjacent to residential areas, and the rapid urbanization in the surrounding areas has led to the inflow of domestic sewage from nearby towns, as well as non-point source pollution and wastewater from agricultural and industrial activities. These anthropogenic factors contribute to the elevated nitrogen and phosphorus concentrations in this river section. Notably, the present study revealed that the TN and total phosphorus TP concentrations in the middle and lower reaches of the Xin River during the winter of 2019 were significantly higher than those recorded in other seasons of the same year. Concomitantly, the biomass and abundance of cyanobacteria in this period attained their annual maxima. These findings collectively confirm that excessive inputs of nitrogen and phosphorus nutrients, driven by anthropogenic activities, can substantially modulate the growth and reproduction of cyanobacteria in aquatic ecosystems.

COD_Mn_ is typically adopted as a comprehensive parameter to represent the pollution extent of organic matter and reductive inorganic matter in aquatic environments. It is also one of the basic evaluation parameters for water eutrophication—higher COD_Mn_ concentrations indicate poorer water quality (Yu et al., 2017). As a key factor influencing the distribution pattern of phytoplankton communities, COD_Mn_ plays a crucial role in the growth and reproduction of phytoplankton. In this study, COD_Mn_ showed an extremely significant positive correlation with both the phylum cyanobacteria and all its dominant species. This phenomenon may be attributed to the fact that organic substances entering the Xin River are oxidized and decomposed into nutrients that can be directly utilized by cyanobacteria. This finding is consistent with the results of studies on other water bodies, such as the Fuhe River (Hu et al., 2023), the Dongjiang River (Jiang et al., 2011), and the Lijiang River (Zhou et al., 2014).

SD characterizes the light transmission capacity of water bodies and serves as a comprehensive index reflecting the amount of suspended solids in water (Qu et al., 2022). Water transparency levels directly impact light intensity within various water layers, and this effect further modulates the photosynthesis of phytoplankton. Different algal species vary in their adaptability to light. For instance, in water bodies with low transparency and significant light limitation, cyanobacteria such as *Microcystis* and *Planktothrix*—which possess gas vesicle buoyancy regulation capabilities (O’Neil et al., 2012; Lenard & Poniewozik, 2022)—and *Phormidium* (a cyanobacterium that can form floating mats or surface flocs in shallow, high-turbidity, and eutrophic waters to utilize the relatively better light conditions at the surface) are the dominant groups. Although the overall transparency of water in study area of the Xin River is relatively low, cyanobacteria like *Microcystis* and *Phormidium* can still reproduce in large quantities and become the dominant species across different survey areas in this reach.

### The hydrology-nutrient-algae cascade mechanism

First, hydrological processes serve as the primary initiating factor of this cascade reaction. In the Xin River basin, intense precipitation during the summer wet season triggers a significant runoff scouring effect. This study indicates that the RSI peaks during this period, reflecting the massive influx of non-point source pollution—such as agricultural runoff and municipal sewage—into the river channel *via* surface runoff. This process aligns with the “nutrient pulse driven by extreme climatic events” theory proposed by Paerl, Hall & Calandrino (2011), which posits that heavy rainfall disrupts the original nutrient equilibrium of rivers by increasing terrestrial inputs. Similarly, in studies of the Poyang Lake hydraulic system, Hu et al. (2023) noted that hydrological fluctuations induced by rainfall are core elements driving the spatiotemporal evolution of nutrients in tributaries like the Fuhe River.

Second, nutrient transport and transformation represent the intermediary stage of the cascade. This study demonstrates that while TN concentrations are higher during the dry season, cyanobacterial biomass peaks during the wet season when TP and COD_Mn_ loadings are elevated. This suggests that in dynamic riverine ecosystems like the Xin River, phosphorus availability is often more limiting than nitrogen, validating the classic assertion by Schindler et al. (2008) regarding the dominant role of phosphorus in freshwater eutrophication.

Furthermore, the highly significant correlation between COD_Mn_ and cyanobacterial biomass suggests that the input of exogenous organic nutrients not only alters the chemical environment of the water but may also accelerate cyanobacterial turnover through microbial cycling (O’Neil et al., 2012). Finally, the cascade culminates in the biological response of the cyanobacterial community. Under conditions of sufficient nutrient supply coupled with high-temperature hydrological environments, cyanobacteria exhibit a distinct competitive advantage. In this study, *Microcystis* and *Anabaena* were observed to be absolutely dominant during the wet season. According to the physiological research by Huisman et al. (2018), these genera possess unique gas vacuoles that allow them to regulate buoyancy and optimize light acquisition in low Secchi depth waters caused by runoff inputs. Moreover, in areas such as the river-lake interface where flow velocity decreases, changes in hydrodynamic conditions (increased residence time) further amplify the bioavailability of nutrients. This transforms these areas into “hotspots” for cascade-driven blooms, a conclusion highly consistent with the “nutrient-water age” synergistic driving mechanism observed by Qu et al. (2022) in Lake Hongze.

## Conclusion

(1) During the survey period, a total of 66 phytoplankton genera belonging to seven phyla were identified in the middle-lower reaches of the Xin River. Chlorophyta, Bacillariophyta, and cyanobacteria constitute the dominant phytoplankton groups in this reach, and the aquatic ecosystem is classified as a Chlorophyta-Bacillariophyta type water body. Cyanobacteria exhibit distinct temporal and spatial distribution patterns. The high-water period in summer represents the primary outbreak period for cyanobacteria, with high-density cyanobacteria cells predominantly concentrated in the water areas of Ruihong Town (located in the main stream) and Xinjangkou (the estuary where the Xin River empties into the lake). *Microcystis*, *Planktothrix*, and *Phormidium* are the main dominant species of cyanobacteria in the main stream, while *Microcystis*, *Phormidium*, and *Anabaena* are the main dominant species of cyanobacteria at the lake entrance.

(2) The water bodies in the middle-lower reaches of the Xin River are generally at a mild eutrophication level, with a relatively high risk of cyanobacterial blooms occurring during the summer wet period and the autumn recession period.

(3) A heterogeneous set of driving factors for cyanobacterial blooms is evident along the middle-lower reaches of the Xin River, indicating considerable spatial variation. TP, PO_4_^3−^-P, COD_Mn_, and SD are the primary factors influencing the distribution of cyanobacterial communities in the main stream region. In contrast, DTN and COD_Mn_ are the key factors affecting the distribution of cyanobacterial communities at the lake entrance region. Nitrogen and phosphorus nutrients are critical determinants of cyanobacterial community distribution in the Xin River. Therefore, controlling the input of exogenous nitrogen and phosphorus, enhancing ecological and environmental protection of the river basin, strengthening aquatic ecological monitoring, and conducting in-depth research on the mechanism of action of influencing factors are of great significance for preventing cyanobacterial blooms in the Xin River.

##  Supplemental Information

10.7717/peerj.21445/supp-1 Supplemental Information 1 Phytoplankton Date

10.7717/peerj.21445/supp-2 Supplemental Information 2 Phytoplankton data and water physicochemical data

## Additional Information and Declarations

### Data Availability

The following information was supplied regarding data availability: The raw data are available in the Supplemental Files.
